# Isolation and Characterization of Functionally Active Extracellular Vesicles from Culture Medium Conditioned by Bovine Embryos In Vitro

**DOI:** 10.3390/ijms20010038

**Published:** 2018-12-21

**Authors:** Krishna Chaitanya Pavani, An Hendrix, Wim Van Den Broeck, Liesbeth Couck, Katarzyna Szymanska, Xiaoyuan Lin, Jenne De Koster, Ann Van Soom, Bart Leemans

**Affiliations:** 1 Department of Reproduction, Obstetrics and Herd Health, Faculty of Veterinary Medicine, Ghent University, Salisburylaan 133, B-9820 Merelbeke, Belgium; KrishnaChaitanya.Pavani@UGent.be (K.C.P.); Jenne.DeKoster@UGent.be (J.D.K.); Ann.VanSoom@UGent.be (A.V.S.); 2 Laboratory of Experimental Cancer Research, Department of Radiation Oncology and Experimental Cancer Research, Ghent University, C. Heymanslaan 10, B-9000 Ghent, Belgium; An.Hendrix@UGent.be; 3 Cancer Research Institute Ghent (CRIG), C. Heymanslaan 10, B-9000 Ghent, Belgium; 4 Department of Morphology-Faculty of Veterinary Medicine, Ghent University, Salisburylaan 133, B-9820 Merelbeke, Belgium; Wim.VandenBroeck@UGent.be (W.V.D.B.); liesbeth.couck@UGent.be (L.C.); 5 Department of Basic Medical Sciences-Physiology group, Faculty of Medicine and Health Sciences, Ghent University, De Pintelaan 185, B-9000 Ghent, Belgium; Katarzyna.Szymanska@UGent.be; 6 Department of Nutrition, Genetics, and Ethology, Faculty of Veterinary Medicine, Ghent University, B-9820 Merelbeke, Belgium; linxiaoyuan23@163.com

**Keywords:** embryo–embryo communication, extracellular vesicles, embryo culture, bovine, Optiprep^TM^ density gradient ultracentrifugation, uptake of embryo-secreted EVs

## Abstract

Extracellular vesicles (EVs) play a possible role in cell–cell communication and are found in various body fluids and cell conditioned culture media. The aim of this study was to isolate and characterize EVs in culture medium conditioned by bovine embryos in group and to verify if these EVs are functionally active. Initially, ultracentrifuged bovine serum albumin (BSA) containing medium was selected as suitable EV-free embryo culture medium. Next, EVs were isolated from embryo conditioned culture medium by OptiPrep^TM^ density gradient ultracentrifugation. Isolated EVs were characterized by nanoparticle tracking analysis, western blotting, transmission, and immunoelectron microscopy. Bovine embryo-derived EVs were sizing between 25–230 nm with an average concentration of 236.5 ± 1.27 × 10^8^ particles/mL. Moreover, PKH67 EV pre-labeling showed that embryo-secreted EVs were uptaken by zona-intact bovine embryos. Since BSA did not appear to be a contaminating EV source in culture medium, EV functionality was tested in BSA containing medium. Individual embryo culture in BSA medium enriched with EVs derived from conditioned embryo culture medium showed significantly higher blastocyst rates at day 7 and 8 together with a significantly lower apoptotic cell ratio. In conclusion, our study shows that EVs play an important role in inter embryo communication during bovine embryo culture in group.

## 1. Introduction

In vivo mammalian embryos develop inside the female reproductive tract (i.e., the oviduct and uterus). When embryos are cultured under in vitro conditions, in the lack of the female genital tract, embryo development is still suboptimal, with lower blastocyst and pregnancy rates [1]. However, preimplantation embryos can support their in vitro development through the secretion of autocrine factors, even without maternal genital tract communication [2,3]. Culturing embryos in groups, and putative secretion of autocrine factors support embryo development and embryo quality in various mammalian species [3,4,5]. The identity of these autocrine factors remains largely unknown. Recently, several proteins that are secreted by embryos of good quality were identified and we demonstrated a beneficial effect of cathepsin during individual culture of bovine embryos [6]. On the other hand, it is very likely that not only proteins are important for communication among embryos (for review see [7]). Moreover, the possible role of extracellular vesicles as a mean for nanomaterial-mediated delivery into gametes and embryos under in vitro conditions recently gained more interest [8,9].

Extracellular vesicles (EVs) are a diverse population of endogenous nano- and micro-sized cell-derived membrane vesicles released by prokaryotic and eukaryotic cells [10]. They contain and transport various bioactive molecules such as mRNAs, miRNAs, proteins [11,12], and lipids [13]. Usually, the nomenclature of extracellular vesicles is associated to their origin and size, in which exosomes (30–150nm) are described as being of endosomal origin and microvesicles (100–1000nm) as being shed from the plasma membrane [14]. Typically, the isolation and identification of EVs are based on specific EV characteristics such as size, morphology, flotation density, and the presence of marker proteins, i.e., Alix, HSP70, and CD9 [15]. Presently, differential ultracentrifugation (UC) is the most common EV isolation method [16]. The ultracentrifugation protocol is quite straightforward. However, this isolation method is time-consuming and may yield EV aggregates after pelleting [17,18]. Recently, it was demonstrated that OptiPrep^TM^ density gradient ultracentrifugation is a reliable EV isolation technique compared to other standard isolation methods such as differential ultracentrifugation and ultracentrifugation and by OptiPrep^TM^ density gradient ultracentrifugation, EVs can be isolated without contamination of other nanoparticles (high density lipoproteins, ribonucleoproteins) [16].

Extracellular vesicles can be isolated from all biological fluids including urine, breast milk, saliva, and serum [19,20]. Since cell culture media are often enriched with fetal bovine serum (FBS), the behavior of in vitro cultured cells can be substantially affected by extracellular vesicles present in FBS [21,22]. Subsequently, a diminished cell growth-promoting activity was observed when the culture medium was prepared using an EV-depleted human serum. Moreover, supplying cells in culture with isolated EVs, isolated from FBS, promoted their growth. Therefore, when analyzing embryo-conditioned media for the presence of EVs, care must be taken to differentiate between embryo-secreted EVs and contaminating EVs derived from a protein source like FBS. Recently, EVs have been implicated as putative messengers for embryo related cross-talk outside the body (i.e., in vitro conditions): Saadeldin et al. [23] showed that in vitro-produced porcine parthenogenetic embryos release EVs in the culture medium. Additionally, Mellisho et al. [24] showed that in vitro-produced blastocysts and parthenogenetic bovine blastocysts can release EVs into the culture medium. Extracellular vesicles isolated from bovine oviduct epithelial cell (BOEC)-conditioned medium enhanced blastocyst quality significantly [25]. In these three studies, EVs were isolated with standard ultracentrifugation techniques. As a consequence, low EV yields were generated, which are operator-dependent. Moreover, as an effect of high-speed ultracentrifugation, EVs can be damaged due to the different applied shear forces [26]. Furthermore, the risk for contamination of EVs by other nanoparticles after standard ultracentrifugation was real [16].

Therefore, the aims of this study were to select initially an EV-free bovine embryo culture medium and optimize a suitable technique for the isolation of EVs from medium conditioned by bovine embryos with a special focus on the purity of EV yields. Furthermore, embryo-released EVs present in culture medium conditioned by bovine embryos were quantified, characterized, and it was investigated whether these EVs were uptaken by zona-intact bovine embryos. Next, embryo culture medium supplements such as bovine serum albumin were assessed for EV contamination or other nanoparticle presence. Finally, the functionality of embryo secreted EVs was determined by supplementing EVs, derived from group embryo culture conditioned medium, to individually cultured bovine embryos.

## 2. Results 

### 2.1. Selection of a Suitable EV-free Embryo Culture Medium for EV Isolation and Characterization after Being Conditioned by Bovine Embryos

Since BSA was considered at the start of this study as a possible EV contamination source, an alternative bovine embryo culture medium was selected based on embryo development and quality parameters. At 45 hpi (hours post insemination), embryos cultured in UC BSA (ultracentrifuged SOF+ITS+BSA; ultracentrifuged synthetic oviductal fluid + insulin-transferrin-selenium + bovine serum albumin), PVP (protein free SOF+ITS+PVP; synthetic oviductal fluid + insulin-transferrin-selenium + polyvinylpyrrolidon), and UC PVP (ultracentrifuged SOF+ITS+PVP) media did not demonstrate any significant difference (*p* > 0.05) in cleavage rate compared with BSA medium (protein containing SOF+ITS+BSA). Similarly, at 7 dpi (days post insemination) and 8 dpi, blastocyst development was not significantly different between BSA (31.54 ± 5.27%, 40.78 ± 3.39%), UC BSA (30.07.1 ± 5.96%, 40.78 ± 6.61%), and in PVP media (28.85 ± 4.04%, 38.67 ± 6.91%; Table 1) while significantly lower blastocyst rates were observed in UC PVP medium (22.97 ± 3.88%, 31.10 ± 4.63%) compared with BSA medium (*p* < 0.05). A similar percentage of blastocysts hatched in UC BSA medium (25.61 ± 13.07 %) and BSA medium (25.36 ± 7.73%) (*p* > 0.05) was observed while significantly fewer blastocysts did hatch in both PVP (15.56 ± 6.83%) and UC PVP (10.20 ± 8.46%) media (Table 1), indicating that the removal of protein decreased hatching rates.

Embryo quality was assessed by scoring three parameters simultaneously: (1) the total cell number (TCN; the sum of trophectoderm cells and inner cell mass), (2) the cell number of ICM (Inner cell mass) compared to TCN (ICM ratio) and (3) the percentage of apoptotic cells (ACR). The TCN and ICM cell number were significantly lower in embryos cultured in UC BSA (TCN: 139 ± 4.33; ICM cell number: 38 ± 1.73), PVP (TCN: 120 ± 5.47; ICM cell number: 31 ± 1.73) and UC PVP (TCN: 107 ± 6.35; ICM cell number: 25 ± 1.42) media compared to BSA medium (TCN: 153 ± 4.89; ICM cell number: 42 ± 3.17) (*p* < 0.05) (Table 2). Moreover, the ACR ratio was significantly higher in PVP (12.4 ± 1.62) and UC PVP (13.1 ± 1.70) media compared to BSA (5.9 ± 0.45) and UC BSA (8.7 ± 0.58) medium (*p* < 0.05) (Table 2).

When EV isolation and characterization experiments were initially performed, it was not clear if BSA was a possible source of EV contamination of the embryo culture medium. In order to avoid any risk and based on the embryo development and quality data, UC BSA medium was selected initially as the most suitable embryo culture medium for EV isolation and characterization after being conditioned by bovine embryos (see above), despite the fact that embryo quality parameters were slightly decreased compared with BSA medium. UC BSA medium was used as embryo culture medium in all the subsequent EV isolation and characterization experiments. For EV functionality experiments (see below), BSA medium was used as culture medium because later on in the experimental process we figured out that BSA was not contaminated by EVs. 

### 2.2. Efficiency of OptiPrep^TM^ Density Gradient Ultracentrifugation for EV Isolation from Bovine Embryo Conditioned Medium

**Figure 1:**
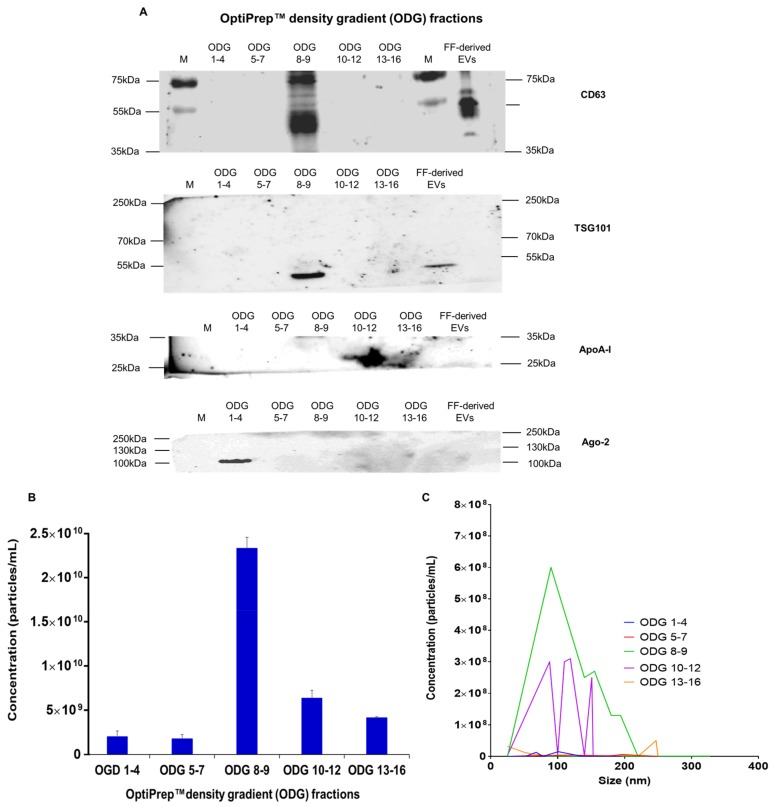
Characterization of EVs derived from concentrated conditioned medium (CCM) to demonstrate the abundance of EVs in the gradient fractions 8 and 9 after OptiPrepTM density gradient (ODG) ultracentrifugation. Pooled gradient fractions (ODG 1–4, 5–7, 8–9, 10–12, 13–16) were analyzed by (**A**) western blot for EV specific markers (CD63, TSG101) and non-EV specific markers (ApoA-I, Ago-2) to determine that the isolation process did not contaminate the EV-rich gradient fraction 8–9. Nanoparticle tracking analysis was applied to measure the (**B**) particle concentration and (**C**) the particle size in all pooled gradient fractions. Abbreviations: ODG 1–4 = OptiPrepTM density gradient fractions 1 to 4 (similar for 5–7, 8–9. 10–12, 13–16), FF derived EVs = follicular fluid derived EVs, M = marker (protein ladder).

Van Deun et al. [16] reported that OptiPrep^TM^ density gradient (ODG) ultracentrifugation is a reliable technique to isolate highly purified EV samples (fraction 8–9). In order to verify the efficiency of this protocol to isolate EVs from bovine embryo conditioned medium (2 mL), pooled fractions (1–4, 5–7, 8–9, 10–12, 13–16; OptiPrep^TM^ density gradient fractions 1 to 4, 5 to 7, 8 to 9, 10 to 12, 13 to 16) were subjected to western blot using EV specific and non-EV specific markers. This analysis showed that only OptiPrep^TM^ density gradient fraction 8–9 was positive for EV marker proteins CD63 and TSG101 (Figure 1A). Non-EV specific marker proteins APO-AI and Ago-2 demonstrated the presence of lipoproteins in fraction 10–12 and ribonucleoproteins in fraction 1–4 (Figure 1A). These observations indicate that EV rich fraction 8–9 was not contaminated by APO-AI and Ago-2 positive nanoparticles. Moreover, nanoparticle tracking analysis showed that the highest particle concentration was measured in the EV rich fraction 8–9 (236.5 ± 1.27 × 10^8^ or 2.36 ± 1.27 × 10^10^ particles/mL) (Figure 1B). Particles were sizing from 25 to 230 nm with an average particle size of 123.7 ± 7.8 nm (Figure 1B,C). Based on these results, EV rich fraction 8–9 was obtained in following experiments to characterize EVs in bovine embryo conditioned and blank BSA medium. 

### 2.3. Bovine Embryos Release Extracellular Vesicles

**Figure 2:**
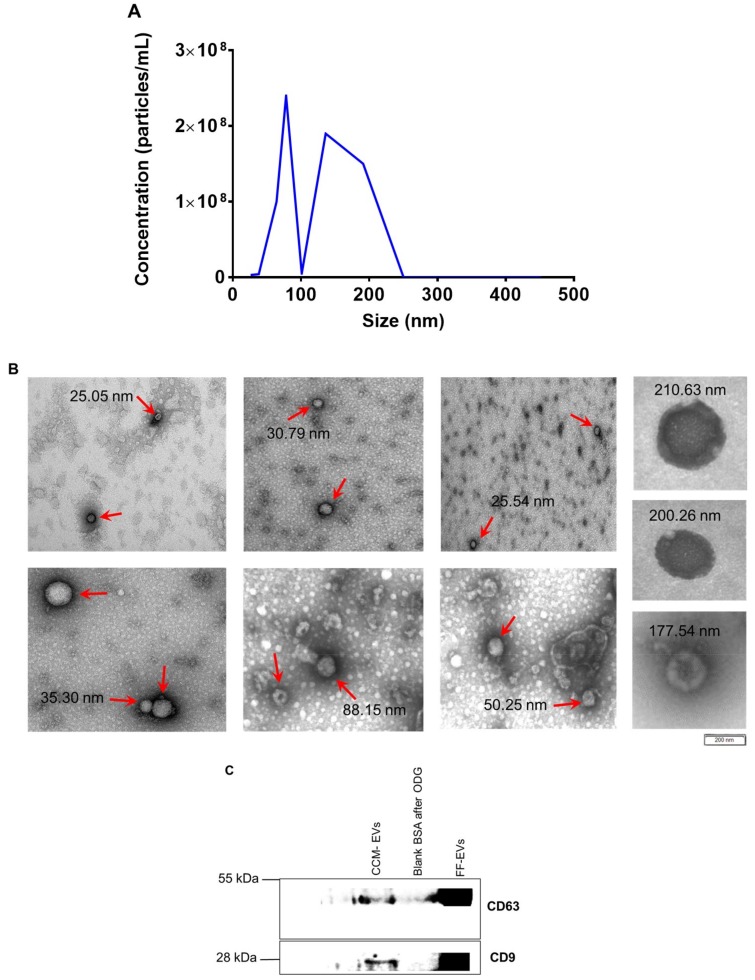
(**A**) Nanoparticle tracking analysis, (**B**) transmission electron microscopy, and (**C**) western blotting were performed to assess the concentration, size, morphology, and presence of EVs isolated from concentrated conditioned medium (CCM). (**A**) The concentration of EVs from 1 mL of bovine embryo conditioned medium (obtained from a pool of 500 embryos) was 40.8 × 10^8^ particles/mL and their size ranged between 25 nm and 250 nm. (**B**) Images demonstrate different EVs indicated with red arrows, size ranging from 25 nm to 250 nm. (**C**) The presence of EVs in OptiPrep™ density gradient (ODG) ultracentrifugated embryo conditioned medium (fraction 8–9); and blank BSA medium after (fraction 8–9) ODG ultracentrifugation was tested using two EV specific (CD63, CD9). EVs isolated from bovine follicular fluid (FF) were included as positive control respectively.

**Figure 3:**
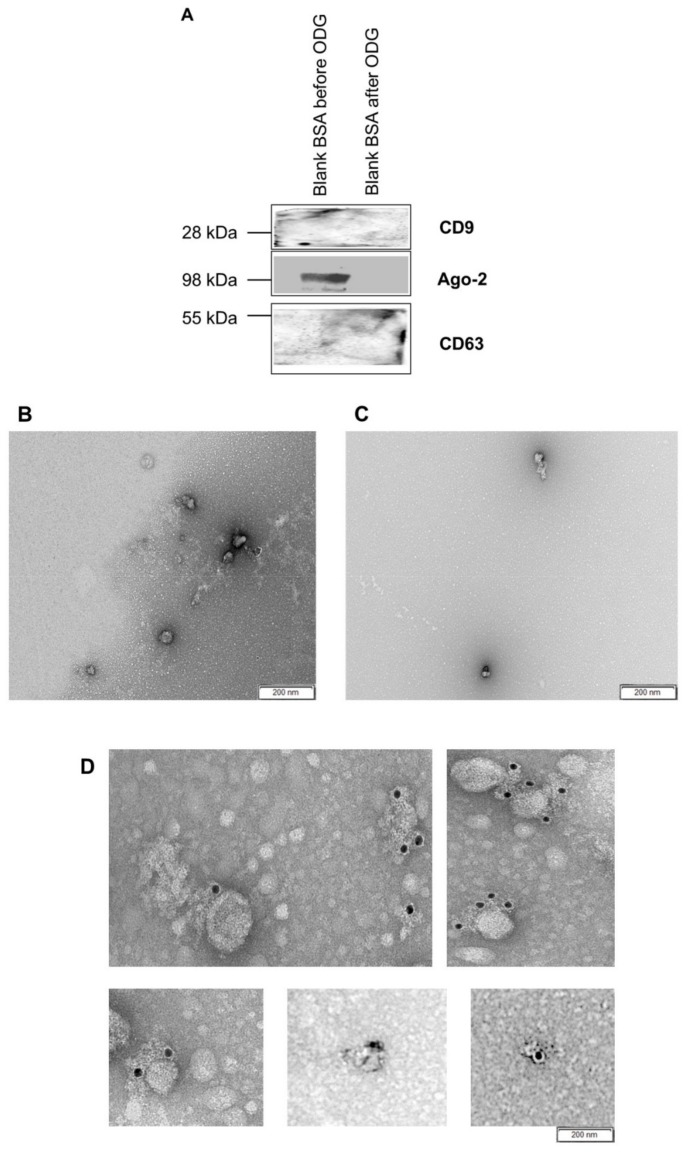
(**A**) The presence of EVs in blank BSA medium before and after (fraction 8–9) ODG ultracentrifugation was tested using two EV specific (CD63, CD9) and one non-EV specific antibodies (Ago-2). Blank BSA medium (SOF+ITS+BSA) was analyzed before (**B**) and after (**C**) OptiPrep™ density gradient (ODG) ultracentrifugation (EVs isolation procedure) by transmission electron microscopy after immunogold labeling with anti-CD63 antibodies. (**D**) EVs isolated from concentrated conditioned embryo culture medium were also analyzed by this method. No membrane-associated gold particles binding was observed in blank BSA medium before and after OptiPrep™ density gradient (ODG) ultracentrifugation (**B**,**C**) but positive immunogold labeling was observed in EVs isolated from concentrated conditioned embryo culture medium. Black dots (10 nm in size) indicate membrane-associated gold particles.

To assess the release of EVs by embryos, presumed zygotes were cultured in UC BSA medium. Moreover, 1 mL of UC BSA conditioned medium appeared to represent a sufficient amount of conditioned medium to perform further analysis. Nanoparticle tracking analysis showed that the concentration of particles from a concentrated pool of 500 embryos was 40.8± 2.72 × 10^8^ particles/mL and particles sizing between 25 and 250 nm (Figure 2A). Moreover, the mean EV size in bovine embryo conditioned medium was 133.8 ± 6.8 nm and the mode was 97.5 ± 19.7 nm. Accordingly, transmission electron microscopy results confirmed the presence of EVs in concentrated conditioned medium (Figure 2B). EVs with bilipid membranes ranging in size from 25 nm to 250 nm were observed. Moreover, transmission electron microscopy after immunogold labeling with anti-CD63 antibodies served as an extra confirmation of the presence of EVs in concentrated conditioned medium (Figure 3D). Western blot analysis demonstrated the presence of three different EV-markers (CD63, TSG 101, and CD9) in concentrated conditioned medium and bovine follicular fluid (Figure 1A and Figure 2C). 

### 2.4. EVs Are Uptaken by Bovine Embryos

**Figure 4:**
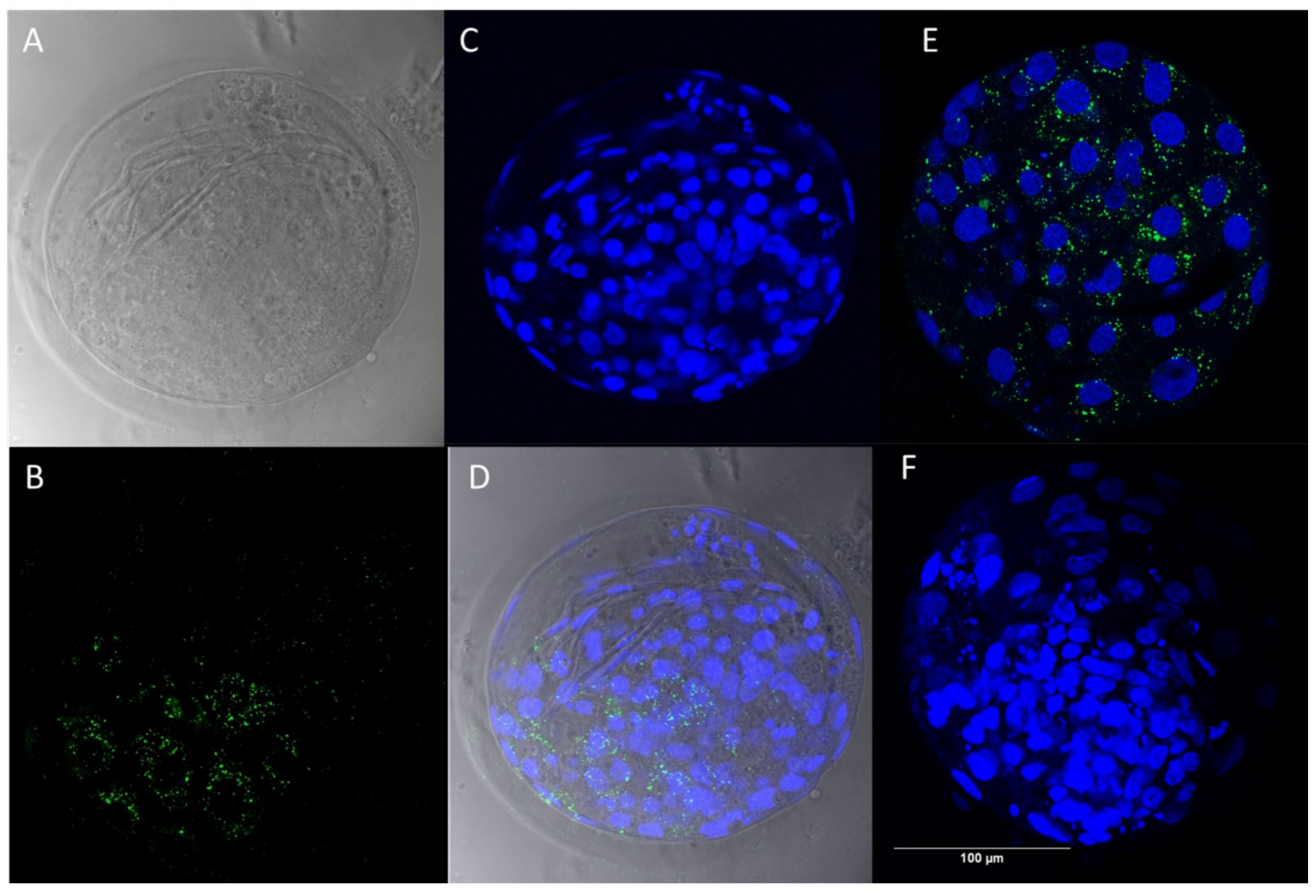
Bright field and fluorescence images demonstrating the passage of EVs through the zona pellucida and the subsequent EVs uptake by the cells of the blastocyst. EVs isolated from concentrated conditioned medium were pre-labeled with green fluorescent PKH67 dye, followed by 0.22 μM filtration) and ultimately co-incubated with 7 dpi bovine embryos in ultracentrifuged SOF+ITS+BSA medium. After co-incubation of (**A**) zona-intact bovine embryos with (**B**) PKH67-labeled EV’s, embryos were washed, fixed, and stained with (**C**) Hoechst 33342 to visualize the nuclei. The (**D**) overlay image demonstrates uptake of green fluorescent-labeled EVs by zona-intact embryos during in vitro embryo culture. (**E**) PKH67-labeled follicular fluid derived EVs (positive control) were also uptaken by bovine embryos in culture. (**F**) PKH67-labeled PBS co-incubated with bovine embryos did not show any green fluorescence (negative control).

By confocal microscopy, it was demonstrated that zona-intact bovine embryos were able to internalize EVs that were isolated from the concentrated bovine embryo conditioned medium. After the release of EVs from cultured embryos, it was observed that PKH67 fluorescently labeled EVs were able to cross the bovine zona pellucida. Moreover, fluorescently labeled EVs were uptaken by exposed in vitro-produced 7 dpi blastocysts (Figure 4A−D; 3D reconstruction as Supplementary File). As a positive control, we showed that green fluorescent labeled EVs were present in embryos co-cultured with EVs isolated from follicular fluid (Figure 4E). No green fluorescent labeling was observed in embryos co-cultured with PBS (Phosphate buffered saline) (labeled with a PKH67 dye, negative control) (Figure 4F).

### 2.5. BSA Containing Bovine Embryo Culture Medium (BSA Medium) Did not Contain EVs but Other Nanoparticles 

**Figure 5:**
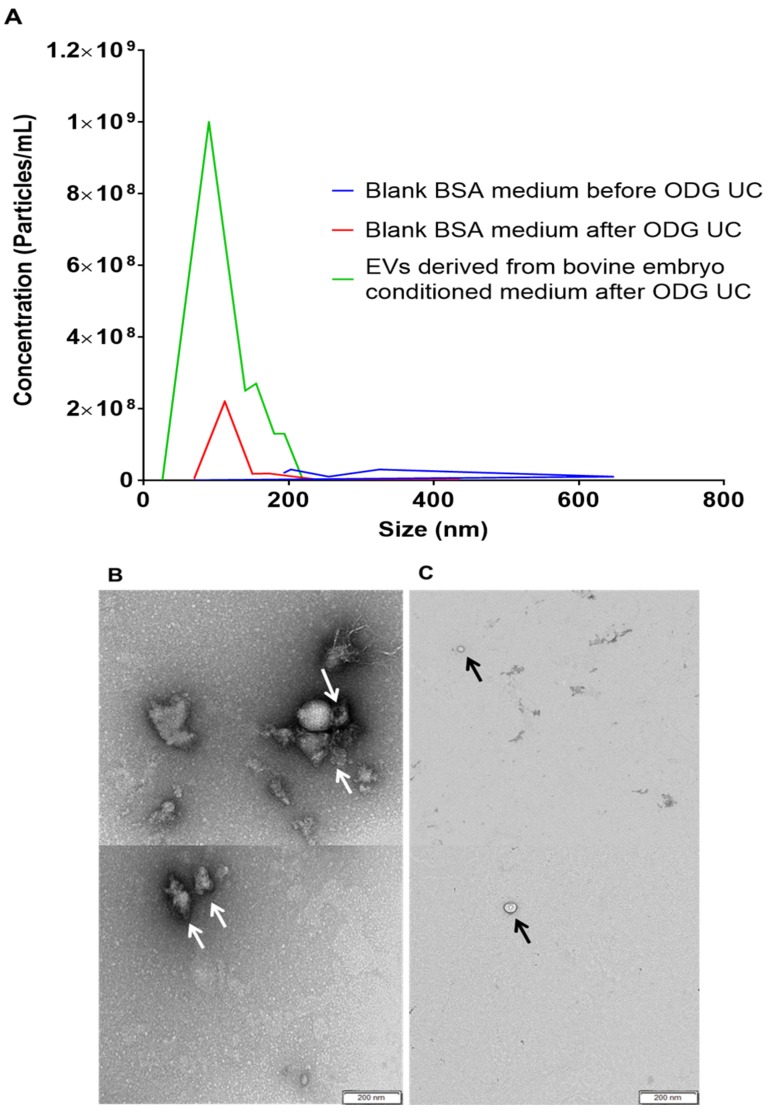
Nanoparticle tracking analysis and transmission electron microscopy were applied to assess the concentration, size and morphology of nanoparticles in blank BSA embryo culture medium (SOF+ITS+BSA) (**A**) before and after OptiPrep™ density gradient ultracentrifugation (ODG UC) (EVs isolation procedure). For the nanoparticle tracking analysis, ODG UC processed embryo conditioned medium (fraction 8–9) was included as a comparison. Similarly, transmission electron microscopy images before (**B**) and after (C) ODG UC (EVs isolation procedure) are shown. The concentration of particles in blank BSA medium before EVs isolation procedure was 66.5 × 10^9^ particles/mL while after EVs isolation procedure the concentration was 53.7 × 10^8^ particles/mL. The nanoparticle size range before EV isolation procedure was between 70 and 400 nm and after EVs isolation procedure between 70 and 250 nm. Images demonstrate (B) EV-like structures (indicated with white arrows) in blank BSA medium before EV isolation. (**C**) After EVs isolation procedure, blank BSA medium did not show EV-like structures anymore but only some lipoproteins (black arrows).

Since fetal bovine serum (FBS) contains EVs [21,22] and BSA medium supported embryo quality better than all other tested media (see Table 2), in this respect, the blank BSA medium was subsequently tested for the presence of BSA-derived EVs before and after OptiPrep™ density gradient ultracentrifugation. Using nanoparticle tracking analysis, a concentration of 66.5 ± 9.25 × 10^9^ particles/mL, sizing between 70 and 400 nm (mean particle size of 139.7 ± 12.6 nm and a mode of 102.7 ± 3.0 nm) was calculated before OptiPrep™ density gradient ultracentrifugation (Figure 5A). After OptiPrep™ density gradient ultracentrifugation, the blank BSA medium demonstrated a concentration of 53.7 ± 1.97 × 10^7^ particles/mL with particles sizing between 70 and 250 nm (mean particle size of 149.1 ± 10.8 nm and a mode of 121.9 ± 5.3 nm) (Figure 5A). In comparison, bovine embryo conditioned medium showed a concentration of 236.5 ± 1.27 × 10^8^ particles/mL with particles sizing between 25 and 230 nm (mean particle size of 123.7 ± 7.8 nm and a mode of 92.8 ± 4.2 nm). In conclusion, particle concentration of blank BSA embryo culture medium before and after the OptiPrep™ density gradient ultracentrifugation showed a significant difference in concentration (Figure 5A). Transmission electron microscopy was subsequently performed to verify the absence of BSA-derived EVs in blank BSA medium. Before OptiPrep™ density gradient ultracentrifugation, blank BSA medium showed EV-like structures (Figure 5B) whereas after OptiPrep™ density gradient ultracentrifugation no EV-like structures were observed. In this latter conditions, only nanoparticles such as lipoproteins and clusters of proteins could be identified (Figure 5C). Based on both nanoparticle tracking analysis and transmission electron microscopy results, it was not clear if BSA did contaminate the culture medium with EVs. 

However, Western blot performed on blank BSA medium before and after OptiPrep™ density gradient ultracentrifugation with EV specific antibodies CD9 and CD63 showed no band formation (Figure 3A) while non-specific EV marker Ago-2 showed only a band for blank BSA medium before OptiPrep™ density gradient ultracentrifugation (Figure 3A). The absence of EVs specific antibody CD63 in blank BSA embryo culture medium (SOF+ITS+BSA) was finally confirmed by immunogold staining (Figure 3B,C). This indicated that blank BSA medium does not contaminate EV preparations with CD9 and CD63 positive EVs. 

### 2.6. Functional Activity of Embryo Culture Medium Derived EVs

In order to verify if EVs released and uptaken by bovine embryos were also able to support embryo development and embryo quality, individual bovine embryo culture in EV enriched BSA culture medium was performed. At 45 hpi (hours post insemination), no significant difference (*p* > 0.05) was observed among all four treatments: group embryo culture in BSA medium (group culture control), individual embryo culture in BSA medium (individual culture control), individual embryo culture in BSA medium + PBS (individual culture control + PBS) and individual embryo culture in BSA medium supplemented with EVs isolated in PBS (EV enriched individual culture). On 7 dpi (days post insemination), no significant difference in blastocyst yield was observed, between group culture control (36.8 ± 1.20%), EV enriched individual culture (34.3 ± 0.09%), and individual culture control + PBS (32.9 ± 0.14%) whereas individual culture control (28.6 ± 0.06%) was significantly different from group culture control (*p* < 0.05) (Table 3). In contrast to group culture control (49.7 ± 1.13%), the blastocyst yield on 8 dpi was significantly lower for individual culture control (40.4 ± 0.08%) and individual culture control + PBS (40.7 ± 0.16%). Interestingly, no significant difference was observed between group culture control and EV enriched individual culture (46.7 ± 0.10%) (*p* > 0.05) (Table 3). Moreover, hatching rates differed significantly between group (25.0 ± 0.92%) and individual embryo culture (individual culture control: 15.3 ± 0.87; individual culture control + PBS: 14.8 ± 1.03; EV enriched individual culture: 14.6 ± 1.09%). These results indicate that EVs present in embryo culture medium support embryo development rates while embryo hatching was not enhanced by the presence of EVs. 

Additionally, embryo quality under similar conditions was assessed by scoring three parameters simultaneously: (1) the total cell number (TCN; the sum of trophectoderm cells and inner cell mass), (2) the cell number of ICM compared to TCN (ICM ratio) and (3) the apoptotic cell number and ratio (ACN/ACR). TCN was not significantly different between group culture control (120.8 ± 49.60), individual culture control + PBS (109.9 ± 30.63) and EV enriched individual culture (109.5 ± 33.30). In contrast, a significantly lower TCN was observed for individual culture control (100.0 ± 26.72) compared to group culture control (*p* < 0.05) (Table 4). Moreover, no differences were observed in ICM ratio between the four different tested culture conditions (group culture control: 31.0 ± 10.50%, individual culture control: 35.5 ± 13.96%, individual culture control + PBS: 30.9 ± 9.42% and EV enriched individual culture: 32.56 ± 10.32%). Interestingly, no significant differences in ACR ratio were observed between EV enriched individual culture conditions (3.3 ± 2.90%) compared to group culture control (5.9 ± 2.39%) while significantly higher ACR rates were observed for individual culture control (8.3 ± 5.69%) and individual culture control + PBS (8.2 ± 4.24%). These results indicate a clear and positive effect of EVs for lowering apoptotic cell ratio in bovine embryos during in vitro embryo culture (Table 4). 

Based on both embryo development and embryo quality data, EV enriched embryo culture medium induced higher embryo development and better quality embryos. Supplementation with EVs did not increase the total cell number, but significantly reduced the ratio of apoptotic cells. This experiment shows that EVs isolated from group culture conditioned medium were functionally active and thus are possible autocrine embryotropins that are missing during individual embryo culture.

## 3. Discussion

In the present study, it was investigated whether EVs that had been reported before to be present in the embryo-conditioned medium were unequivocally resulting from embryonic secretions or could also be possible contaminants from the culture media that were being used. In order to remove putative EVs beforehand from the culture medium, replacement of BSA by PVP had an obvious negative effect on bovine embryo development. Since embryo development was not different between BSA and UC BSA medium; and embryo quality was only slightly decreased, UC BSA medium was initially selected in this study as a suitable embryo culture medium for EV isolation and characterization after being conditioned by bovine embryos. OptiPrep^TM^ density gradient ultracentrifugation showed to be a reliable technique to isolate EVs from the bovine embryo conditioned medium. An EV rich sample was isolated from fraction 8–9. It was demonstrated that bovine embryos cultured in UC BSA medium can release EVs. Besides, these EVs were able to cross the intact zona pellucida and subsequently were internalized by bovine embryonic cells during embryo culture. Ultimately, we demonstrated that blank BSA embryo culture medium was not a source of contaminating EVs suggesting that blank BSA medium could be used as well as EV-free embryo culture medium to test EV functionality. After individual embryo culture in BSA medium enriched with isolated EVs embryo development and quality rates were significantly enhanced.

In the current study, it was demonstrated that in contrast to BSA medium, embryos cultured in PVP medium had a lower blastocyst development and quality. Similarly, Kato and Nagao [27] demonstrated that PVP medium had an adverse effect on the bovine embryo. They injected PVP medium into embryos at the pronuclear stage and observed a lower cleavage rate and blastocyst formation. Moreover, a reduction in total cell number and increased apoptotic cell ratio was demonstrated. It was suggested by these authors that the reduced cells numbers in blastocysts were due to the PVP macro-molecule. It was shown that this macro-molecule induced deterioration of microfilaments. Since microfilaments are essential structures in mitosis and cytokinesis, their demise could impact embryo development [28]. Additionally, when embryos were obtained from bovine oocytes matured with PVP enriched maturation medium, lower blastocyst rates and an increased apoptotic index were observed [29,30]. Based on these facts, it can be hypothesized that PVP and UC PVP media are not the preferred choice to culture bovine embryos in vitro.

The alternative approach to get rid of eventual contaminating EVs was to ultracentrifuge the medium. It was shown that bovine embryos were able to develop in ultracentrifuged BSA medium (UC BSA) to the same rate as BSA medium. However, the TCN and ICM cell numbers were significantly lower in embryos produced in UC BSA medium compared with BSA medium. It can be hypothesized that some necessary protein or other supplements in the SOF+ITS+BSA medium were removed during ultracentrifugation. Therefore, we decided to continue with BSA medium for functional assays, since this medium is routinely used in bovine embryo culture. 

This study demonstrated that OptiPrep™ density gradient ultracentrifugation is a reliable method to isolate an EV rich fraction (8–9), without contamination of other nanoparticles, from bovine embryo conditioned culture medium (UC BSA medium). Van Deun et al. [16] showed previously that EV-associated proteins and RNAs were better conserved after OptiPrep™ density gradient ultracentrifugation than differential ultracentrifugation and commercially-available precipitation kits. In agreement with the results from this study, Van Deun et al. [16] also reported that OptiPrep™ density gradient ultracentrifugation is preferred over other EV isolation ultracentrifugation techniques because EV rich samples can be harvested without contamination by other nanoparticles such as lipoproteins and ribonucleoproteins. 

Our results showed that EVs can be isolated from ultracentrifuged SOF+ITS+BSA concentrated medium conditioned by bovine embryos. Transmission electron microscopy and western blotting results showed the presence of EVs in bovine embryo culture medium. Moreover, nanoparticle tracking results confirmed that a majority of the EVs display a size between 25 and 250 nm. This result is in agreement with Saadeldin et al. [23], as they have demonstrated in porcine embryos that the EV size varies based on the developmental stage of the embryo (two-cell until the blastocyst stage: EVs diameter < 30 nm; blastocyst and hatching stage: <200 nm). On contrary, Mellisho et al. [25] has demonstrated that the diameter of EVs released by bovine blastocysts was sizing between 30 and 385 nm. This latter finding is unexpected since some of these vesicles are larger than the pores of the zona pellucida in bovine embryos, which are on average between 150 and 220 nm in diameter, depending on the embryonic stage [31,32]. Hence one would expect that because of physical limitations EVs larger in diameter than 250 nm cannot be released into the medium. If this assumption is correct, it would be logical that the size of embryonic EVs is correlated to the embryonic stage, as a consequence of the pore size of the zona pellucida at each embryonic stage [31]. As a consequence, apoptotic bodies (500–5000 nm) [9,10,11] released by degenerated embryos in group will not reach the culture medium or influence other healthy embryos.

This study also showed that EVs released to the culture medium by bovine embryos and isolated from concentrated conditioned medium were incorporated by embryonic cells. By labeling the EVs with the PKH67 dye and subsequent co-incubating them with in vitro-produced bovine embryos, it was demonstrated that EVs were passing through the zona pellucida and subsequently uptaken by the embryonic cells of the bovine blastocyst. In general, the permeability of the zona pellucida for specific molecules depends on the size of the molecule and the physicochemical properties, such as hydrophilic–lipophilic interactions. In particular, lipid-containing molecules, and by extension also EVs, which are membranous structures, penetrate the zona pellucida easier than hydrophilic molecules [33]. To internalize with the cells, EVs are absorbed by the cell surface. Then, the EV plasma membrane fuses with the cells and as a consequence their contents are released into the cytosol of the cells. It has also been suggested that intact EVs enter the cells as such [34]. Similarly to these data, Saadeldin et al. [23] demonstrated that EVs derived from porcine embryos were uptaken by cloned embryos. Moreover, Almiñana et al. [35] reported the uptake of EVs derived from bovine oviduct epithelial cells by in vitro produced bovine embryos. 

In this study, it is demonstrated for the first time that BSA added to embryo culture medium is not a possible source of EVs, but other factors of BSA are supporting embryo development and quality. During ultracentrifugation some other necessary factors (possibly transferrin, which has a higher MW than insulin or other unknown factors) were lost during this procedure, leading to the lower quality embryos cultured in UC BSA medium. EV-like structures or other nanoparticles were also identified in blank BSA medium before ultracentrifugation. Stolk and Seifer [36] identified similar EV-like structures in bovine serum albumin solution (Sigma A8412). They termed them as ‘sham’ vesicles that were interfering with the functional activity of extracellular vesicles isolated from mesenchymal stromal cell culture medium. These ‘sham’ vesicles were absent in ultracentrifuged blank BSA medium. Additionally, evidence was provided by immunogold staining and WB that these vesicles were different from extracellular vesicles. It also indicated that the isolation procedure [16] could remove all unnecessary vesicles or nanoparticles compared to regular differential ultracentrifugation methods [37]. Even though BSA is not the source of EVs, UC BSA medium was used for culturing bovine embryos to avoid any other anomalies. 

Ultimately, this is the first study that demonstrates that EVs are embryotropins and thus functionally active particles. By supplementing EVs, isolated from culture medium conditioned by bovine embryos, to individual embryo culture medium an enhanced effect on embryo development and quality was observed as reduction of embryo cell apoptosis. Wydooghe et al. [3] showed that group culture is able to rescue the development of slow-cleaving embryos. Based on our observations, we understand now why embryo culture in group favors individual embryo culture in terms of embryo development and quality [3,7]. In conclusion, this study showed that the superior embryo development and quality in group culture conditions is at least partly due to the released and uptaken EVs by bovine embryos in group. The autocrine embryotropin role demonstrated by EVs is likely due to the transmission of EV carried proteins, mRNA, and microRNA [7]. Interestingly, higher hatching rates were observed in group culture conditions compared to individual culture conditions regardless of EVs were supplemented to the individual culture conditions. This indicates that blastocyst hatching occurs independently from EVs in the culture medium. According to Kaaekuahiwi et al. [38] an embryo in culture produces plasminogen activator, a protease that can activate plasminogen, which creates enzyme plasmin that weakens the zona pellucida to simplify hatching. Based on the fact that in group culture 25 embryos per 50 µL medium were cultured while in all individual embryo culture conditions one embryo per 20 µL medium was cultured we hypothesize that a higher concentration plasminogen activator is present under group conditions compared to individual culture conditions. This justifies higher hatching rates in group culture than individual embryo culture. 

## 4. Material and Methods

### 4.1. Media and Reagents

Tissue culture media (TCM)-199-medium, minimal essential medium (MEM) non-essential amino acids (100×), synthetic basal medium eagle amino acids, gentamycin, and kanamycin were purchased from Life Technologies Europe (Ghent, Belgium). Phosphate-Buffered Saline (PBS) was obtained from Gibco™ 20012019, Thermo Fisher Scientific, (Waltham, MA, USA). All other chemicals not otherwise listed were obtained from Sigma-Aldrich (Diegem, Belgium). All media were filtered before use (0.22 μM Pall Corporation, Ann Arbor, MI, USA). 

### 4.2. In Vitro Embryo Production

Routine in vitro methods were used for bovine embryo production, as described previously by Wydooghe et al. [39]. Briefly, at the local abattoir cow ovaries were obtained and processed within 2 h after collection. On arrival at the lab, the ovaries were washed three times in warm physiological saline (with kanamycin (25 mg/mL)). Cumulus-oocyte complexes (COCs) were aspirated from antral follicles sizing between 4 and 8 mm diameter using an 18-gauge needle attached to a 10 mL syringe. Subsequently, only highly viable oocytes with uniformly granulated cytoplasm and surrounded by more than three compact layers of cumulus cells were cultured in groups of 60 COCs in 500 mL modified bicarbonate buffered TCM-199 (supplemented with 50 mg/mL gentamicin and 20 ng/mL epidermal growth factor) in 5% CO_2_ in air for 22 h at 38.5 °C. 

Frozen-thawed bull spermatozoa were separated using a 45/90% percoll^®^ gradient (GE Healthcare Biosciences, Uppsala, Sweden). Next, the sperm pellet was washed in IVF–Tyrode’s albumin–pyruvate–lactate (TALP) medium, containing bicarbonate-buffered Tyrode solution. Then, the sperm concentration was adjusted to a final sperm concentration of 1 × 10^6^ spermatozoa/mL using IVF–TALP medium enriched with BSA (Sigma A8806; 6 mg/mL) and heparin (25 mg/mL).

After 22 h maturation, bovine oocytes were washed in 500 µL IVF-TALP and subsequently co-incubated with Percoll^®^ washed bull spermatozoa. After 21 h gamete co-incubation, presumed zygotes were vortexed to remove surplus zona attached cumulus and sperm cells. The presumed zygotes were cultured in groups of 25 in 50 µL droplets synthetic oviductal fluid enriched with non-essential and essential amino acids (SOFaa), ITS (5 µg/mL insulin; 5 µg/mL transferrin; 5 ng/mL selenium) and medium droplets were covered with mineral oil and incubated at 38 °C in 5% CO_2_, 5% O_2_ and 90% N_2_. Depending on the experimental set-up, the culture medium was supplemented either with 4 mg/mL bovine serum albumin (BSA) (Sigma A9647) or 0.1 mg/mL polyvinylpyrrolidone (PVP) (Sigma P5288). In some experiments, presumed zygotes were transferred to ultracentrifuged SOF+ITS+BSA/PVP medium to prevent all possible contamination with EVs. In the final experiment, testing the possible functional role of EVs in group embryo culture, the presumed zygotes were cultured either in groups of 25 embryos in drops of 50 µL or individually in drops of 20 µL culture medium supplemented with 4 mg/mL BSA (SOF+ITS+BSA medium). For individual culture conditions, 10 mL of three different media were prepared in order to wash and culture presumed zygotes: (1) 10 mL SOF+ITS+BSA medium (individual culture control), (2) 10 mL SOF+ITS+BSA medium + 25 µL PBS (individual culture control + PBS; 1/400), and (3) 10 mL SOF+ITS+BSA medium + 25 µL PBS containing 0.228 µg/mL isolated EVs (EV enriched individual culture). All media were filtered before use (0.22 μM Pall Corporation, Ann Arbor, MI, USA) and culture media drops were subsequently covered with mineral oil (SAGE™ oil for tissue culture, ART-4008-5P, a CooperSurgical Company, Målov, Denmark) and incubated at 38 °C in 5% CO_2_, 5% O_2_ and 90% N_2_. 

At 45 h post insemination (hpi), embryo cleaving was scored as the percentage of cleaved embryos out of presumed zygotes. At seven and eight days post-insemination (dpi) blastocyst formation was calculated as the percentage of blastocysts out of presumed zygotes. By comparing the number of hatching/hatched blastocysts with the total number of blastocysts, the hatching rate at 8 dpi was obtained. 

Protein containing (SOF+ITS+BSA) and protein free (SOF+ITS+PVP) media will be further referred to as BSA and PVP media respectively. Similarly, ultracentrifuged SOF+ITS+BSA and ultracentrifuged SOF+ITS+PVP embryo culture media will be referred to as UC BSA and UC PVP media respectively. 

### 4.3. Embryo Quality Assessment

At 8 dpi, the identified blastocysts were split into four groups according to the experiments: culture medium (BSA, UC BSA media and PVP, UC PVP media) and culture conditions (group culture control, individual culture control, individual culture control + PBS, EV enriched individual culture) and fixed for 20 min in 2% paraformaldehyde (*w*/*v*). Subsequently, embryo quality, assessed by a differential apoptotic staining, was scored by a double-immunofluorescent staining against CDX2 and active caspase-3 [40]. CDX2 is a transcription factor which is only expressed in the cells of the trophectoderm while active caspase-3 has a central role in the apoptotic pathway. Moreover, Hoechst was used to stain all the nuclei of the embryos. This staining protocol allowed instantaneous evaluation of three crucial parameters of embryo quality, i.e., the total cell number (TCN), the proportion of inner cell mass (ICM) relative to the TCN (ICM ratio), and the apoptotic cell ratio (ACR), the percentage of apoptotic cells relative to the TCN.

### 4.4. Preparation of EV-Depleted BSA Embryo Culture Medium 

According to the literature [21,22], fetal bovine serum (FBS) contains EVs, and by extrapolation, bovine serum extracted BSA may also contain EVs. To eliminate possible EV contamination it is necessary to ultracentrifuge the culture medium before the start of embryo culture [21]. So, the BSA medium (embryo culture medium) and HEPES-TALP+BSA (washing medium) were subjected to ultracentrifugation at 4 °C for 18 h at 100,000× *g* [21]. This protocol was also applied to PVP medium. In anticipation to the final experiment testing the functionality of isolated EVs in group culture medium, we showed that ultimately BSA was not a source of EV contamination. Therefore, we did not ultracentrifuge the BSA culture medium anymore for this experiment.

### 4.5. Concentration of UC BSA Culture Medium Conditioned by Bovine Embryos

Culture medium conditioned by bovine embryos was collected, pooled, and concentrated in preparation for EV isolation. Per embryo culture replicate, a group of 100 presumed zygotes was cultured in ultracentrifuged SOF+ITS+BSA culture medium (UC BSA medium; 25 zygotes per 50 µl droplet), and on 8 dpi 200 µL embryo conditioned medium was collected and stored in −80 °C. To pool 2 mL embryo conditioned culture medium to perform one OptiPrep™ density gradient ultracentrifugation experiment, 10 replicates were performed with a total of 1000 presumed zygotes. For EV characterization, uptake, and functionality experiments, 1 mL pooled embryo conditioned medium provided sufficient EV yields. For this experiment, five replicates with a total of 500 cultured presumed zygotes were performed in order to obtain a 1 mL pooled medium. 

**Scheme 1:**
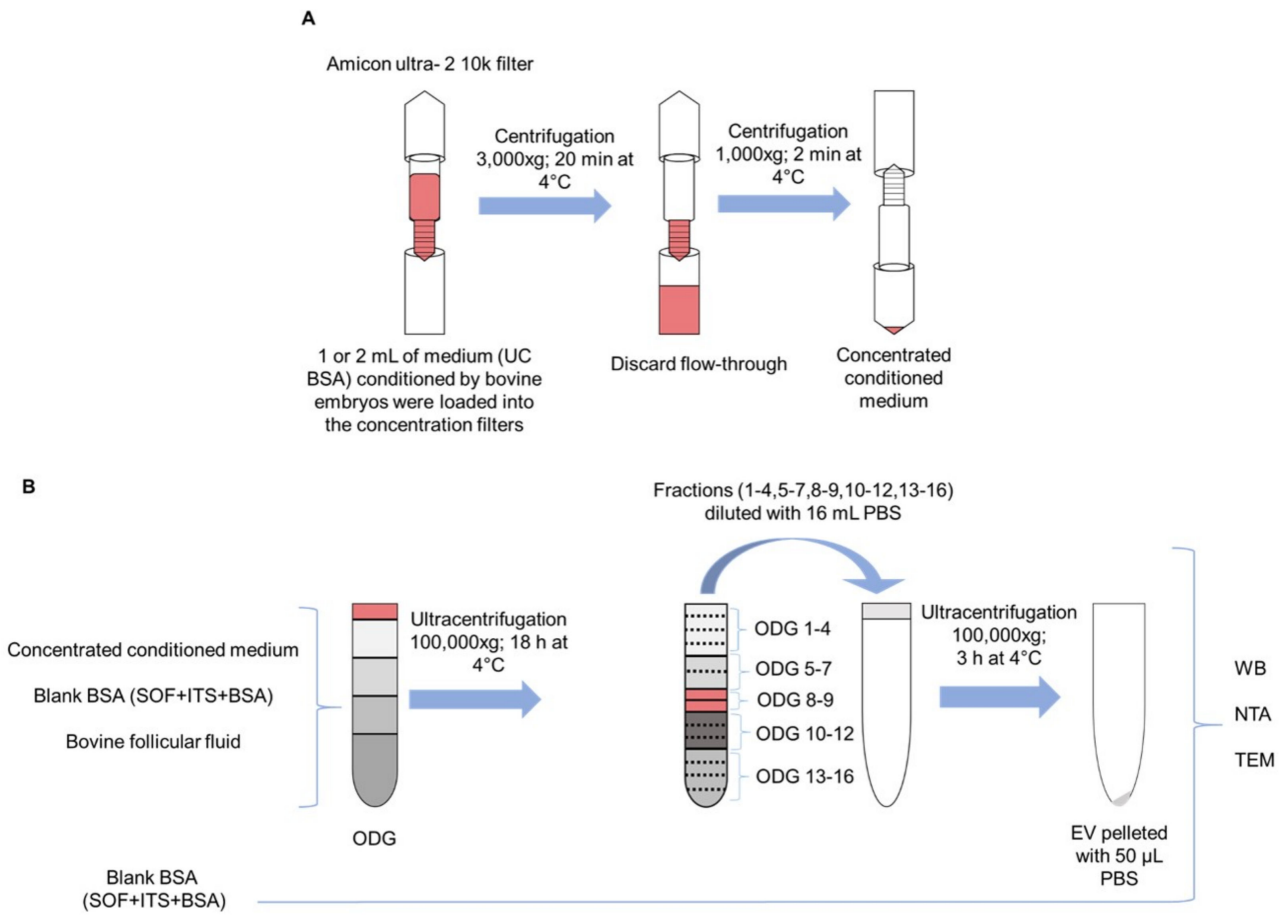
A schematic overview shows the (**A**) preparation of EV-depleted embryoculture medium and concentration of EV rich fraction; and (**B**) OptiPrep^TM^ density gradient (ODG) ultracentrifugation protocol to isolate EVs in this study. Based on the experimental design either 1 mL or 2 mL bovine embryo conditioned medium was subjected to the concentration step before ODG ultracentrifugation. A four layer gradient was established by layering 4 mL of 40% OptiPrep™ gradient solution, 4 mL of 20%, 4 mL of 10%, and 3.5 mL of 5% solutions respectively, with a total of 16 fractions (each fraction is 1 mL) including the sample. Abbreviations: WB: western blotting; NTA: nanoparticle tracking analysis; TEM: transmission electron microscopy.

To concentrate EVs in the pooled embryo conditioned medium samples, 1 or 2 mL conditioned culture medium was loaded in Amicon Ultra-2 10 k centrifugal filters (UFC201024, Merck Millipore, Billerica, MA, USA) and centrifuged at 4 °C for 10–20 min at 3000× *g* using a swinging bucket rotor. The concentration of the samples was achieved by upside-down centrifugation at 4 °C for 2 min at 1000× *g*. The eluates were retrieved from the flow-through reservoir (Scheme 1A). Concentrated conditioned medium and blank BSA medium samples were subjected to EV isolation and quantification. As a control, the blank BSA medium that did not undergo EV isolation was used for characterization.

### 4.6. EV Isolation by OptiPrep^TM^ Density Gradient

OptiPrep™ density gradient (ODG) ultracentrifugation was conducted as previously reported by Van Deun et al. [16]. Briefly, appropriate amounts of a homogenization buffer (10 mM Tris-HCl (Tromethamine - Hydrochloric acid), 1 mM EDTA (Ethylenediaminetetraacetic acid) and 0.25 M sucrose (pH 7.4)) and an iodixanol working solution were mixed in order to prepare 5, 10, 20, and 40% iodixanol solutions. The iodixanol working solution was made by adding a working solution buffer (60 mM Tris-HCl, 6 mM EDTA, 0.25 M sucrose (pH 7.4)) to a stock solution of OptiPrep™ (60% (*w*/*v*) aqueous iodixanol solution). Moreover, the gradient was prepared in a 16.8 mL open-top polyallomer tube (Beckman Coulter) by layering 4 mL of 40%, 4 mL of 20%, 4 mL of 10%, and 3.5 mL of 5% solutions on top of each other. Concentrated conditioned medium (as explained before, UC BSA culture medium conditioned by bovine embryos was collected on 8 dpi and pooled until 1 or 2 mL and subsequently concentrated) was overlaid onto the top of the gradient. Subsequently, the gradient was centrifuged at 4 °C for 18 h at 100,000× *g* (SW 32.1 Ti rotor, Beckman Coulter, Brea, CA, USA). Then, all 16 gradient fractions were divided into six samples by pooling the fractions 1–4, 5–7, 8–9, 10–12, 13–16 respectively. The pooled fractions were added to 14 mL PBS. Subsequently, the separate suspensions were centrifuged at 4 °C for 3 h at 100,000× *g*. The resulting pellets were resuspended in 50 µL PBS and stored at −80 °C for further EV characterization. Out of 16 layers of gradient fractions, EVs were mostly found in fraction 8 and 9 (as previously reported by Van Deun et al. [16]). For subsequent characterization of EVs from culture medium conditioned by bovine embryos, both fractions were pooled, diluted in PBS, centrifuged, resuspended, and stored as described above. All relevant data on the procedures of EV isolation and analysis have been submitted to EV-TRACK knowledgebase (http://evtrack.org/review.php, accessed on 30 August 2018) EV-TRACK ID: EV180007 (EV-METRIC 87%) [41]. This isolation procedure was repeated with blank BSA medium and bovine follicular fluid (positive control) (as detailed in Scheme. 1B)

### 4.7. EV Characterization

#### 4.7.1. Nanoparticle Tracking Analysis

The NanoSight LM10 microscope (Malvern Instruments Ltd., Malvern, UK) was initially used to measure the size and concentration of the particles from pooled OptiPrep^TM^ density gradient fractions (1–4, 5–7, 8–9, 10–12, 13–16). Subsequently, the EV rich fractions (8–9) isolated from concentrated blank BSA and bovine embryo conditioned culture medium; and BSA blank medium before ODG ultracentrifugation was subjected to nanoparticle tracking analysis. For each individual sample, three videos of 60 s were recorded and analyzed with detection threshold 3 and camera level 13. All videos were analyzed by NTA Software version 3.2. To achieve optimal measurements, all EV samples were diluted with PBS to obtain a particle concentration within the optimal range (3 × 10^8^—1 × 10^9^) of the NTA Software.

#### 4.7.2. Transmission Electron Microscopy

Blank BSA and concentrated conditioned media were subjected to transmission electron microscopy for the identification of EVs. Each sample was deposited on precoated formvar/carbon support film copper mesh electron microscopy grids (FCF200H-CU-TB; Aurion, Netherlands). Grids were labeled for 45 s with 1% uranyl acetate (in double-distilled water). Prepared grids were examined using electron microscopy (JEM 1400 plus, JEOL, Benelux; and Ziess EM 109, Carl Zeiss, Jena, Germany). Moreover, images were made by Quemasa charge-coupled device camera (Olympus Soft Imaging solutions GMBH, Munster, Germany).

#### 4.7.3. Immunoelectron Microscopy

Immunogold labeling was performed with some minor modifications as previously described by Van Deun et al. [16]. Briefly, extracellular vesicles isolated from concentrated conditioned medium and presumed extracellular vesicles from blank BSA medium were attached to Formvar carbon-coated grids. Subsequently, the grids were incubated for 1 h in 0.1% BSA in PBS blocking solution. After blocking, the grids were rinsed five times with BSA-c buffer (0.1% BSA-c + PBS, pH 7.4). Later on, the grids were exposed for 1 h to primary CD63 mouse antibodies (1:50 in BSA-c buffer, ab8219, Abcam, Cambridge, UK). Then, the grids were washed five times with BSA-c buffer. After these wash steps, the grids were subsequently incubated for 30 min with secondary rabbit anti-mouse IgG (Immunoglobulin G) antibodies (1:500 in BSA-c buffer, Zymed, San Francisco, CA, USA) together with protein A-gold complex sizing 10 nm (1:40 in BSA-c buffer, Cell Microscopy Core, Cell Biology, University Medical Center Utrecht, Utrecht, The Netherlands). Additionally, blocking efficiency was assessed by performing the immunogold labeling protocol in the absence of primary antibodies. Then, the grids were labeled with neutral uranyl acetate, embedded in methylcellulose/uranyl acetate and analyzed with a Tecnai Spirit transmission electron microscope (FEI, Eindhoven, The Netherlands). Images were obtained with a Quemasa charge-coupled device camera (Olympus Soft Imaging solutions GMBH, Munster, Germany). 

#### 4.7.4. Western Blotting

To test the presence of EVs and other nanoparticles in all pooled gradient fractions (1–4, 5–7, 8–9, 10–12, 13–16) harvested after ODG ultracentrifugation, western blot was performed with EV specific (CD63 and TSG101) or non-EV specific (Ago-2 and Apo-AI) markers. Subsequently, the EV rich fractions (8–9) isolated from concentrated blank BSA, bovine embryo conditioned culture medium and bovine follicular fluid were subjected to western blot to identify EVs by EV specific markers (CD63 and CD9). All samples were suspended in a reducing buffer (0.005% bromophenol blue, 3% 2-mercaptoethanol, 9.2% SDS, 40% glycerol and 0.5 M Tris-HCl (pH 6.8)) and boiled for 5 min at 95°C. Protein samples were separated by SDS polyacrylamide gel electrophoresis and subsequently transferred to a nitrocellulose membrane (Bio-Rad, Hercules, CA, USA). Next, the membrane was blocked at room temperature with 5% skim milk TBST for 45 min. Subsequently, the membranes were exposed to CD63 rabbit (1:200 in 5% BSA + 0.5% Tween PBS ab68418, Abcam, Cambridge, UK), TSG101 mouse (1:1000 in 5% milk + 0.5% Tween PBS, sc-7964, Santa Cruz, CA, USA), Ago-2 rabbit (1:1000 in 5% milk + 0.5% Tween PBS, ab32381, Abcam, Cambridge, UK), ApoA-I mouse (1:1000 in 5% milk + 0.5% Tween PBS, sc-376818, Santa Cruz, CA, USA), and CD9 rabbit (1:1000 in 5% BSA + 0.5% Tween PBS, CST- D3H4P, Cell Signaling Technology, Boston, MA, USA) primary antibodies at 4 °C. After overnight incubation, the membranes were extensively washed with 0.5% Tween in PBS. Then, the membranes were incubated with the appropriate secondary antibodies (anti-mouse IgG (GE Healthcare, UK), 1:3000 in 5% milk + 0.5% Tween PBS; anti-rabbit IgG (GE Healthcare, Buckinghamshire, UK), 1:4000 in 5% BSA + 0.5% Tween PBS. After a final wash step, chemiluminescence substrate (WesternBright Sirius, Advansta, Menlo Park, CA, USA) was added to the membranes. Imaging was performed using Proxima 2850 Imager (IsoGen Life Sciences, De Meern, The Netherlands). 

### 4.8. EV Labeling, EV Embryo Co-Incubation, and EV Uptake

Extracellular vesicles isolated from 1 mL culture medium conditioned by bovine embryos and bovine follicular fluid (positive control) were attached to a green fluorescence lipophilic dye (PKH67, Sigma, Brussels, Belgium) after sample concentration. The protocol was based on Almiñana et al. [35] including a few modifications. First, an EV suspension was made by adding 25 μL concentrated EV sample (20.4 × 10^8^ particles per ml) to 125 μL diluent C (CGLDIL: diluent C used for general membrane labeling). Along with the preparation of the EV sample, a negative control was included by adding 25 μL PBS to 125 μL diluent C. The dilution of the dye was obtained by mixing 1 μL dye (P7333, PKH67 linker) with 250 μL diluent C (1:250 dilution). From the dye dilution, 125 μL mixture was added to EV and negative control suspensions and incubated at room temperature for 5 min (final dye concentration: 5 × 10^−6^ M). To stop the labeling reaction, one ml of EV-free FBS (ultracentrifuged FBS at 4 °C for 18 h at 100,000× *g*) was added to the suspensions. The EV suspension tube was filled with M199 medium + 5% EV-free FBS and twice ultracentrifuged at 100,000× *g* for 30 min at 4 °C to remove the dye excess. The resulting pellet containing PKH67-labeled EVs was resuspended in 100 μL UC BSA embryo culture medium (5.2 × 10^8^ particles per ml). At 7 dpi, in vitro-produced embryos were transferred to fresh 50 µL droplets UC BSA medium containing PKH67-labeled EVs (25 embryos/droplet) or incubated with the control suspension (PKH67–labeled PBS). After 18–20 h incubation, embryos were washed two times using an EV-free medium to remove any extraneous labeled EVs not internalized by the embryonic cells and subsequently fixed at room temperature for 30 min with 4% paraformaldehyde. These embryos were stained with Hoechst 33342 (dilute 1:100 PBS) for 10 min and imaged using a Leica TCS-SP8 X confocal microscope (Leica Microsystems, Wetzlar, Germany). This experiment was repeated four times and 15–20 embryos were assessed per replicate.

### 4.9. Experimental Design: Initial Selection of a Suitable Embryo Culture Medium for EV Isolation and Characterization after Being Conditioned by Bovine Embryos

In order to select a suitable embryo culture medium for EV isolation and characterization after being conditioned by bovine embryos, alternative media were evaluated based on embryo quality and development. In a first approach, BSA was replaced by polyvinylpyrrollidone (PVP) for embryo culture to remove all protein sources from the culture medium, which were possibly containing EVs. Additionally, to evaluate whether ultracentrifugation of BSA medium (to deplete possible putative contaminating like EVs) was affecting embryo development, the presumed zygotes (*n* = 1899, 8 replicates) were allocated to four different culture media (BSA, UC BSA media, and PVP, UC PVP media). For BSA and PVP media, the presumed zygotes were washed with standard HEPES-TALP+BSA before in vitro culture; whereas with UC BSA and UC PVP media, the presumed zygotes were washed three times by ultracentrifuged HEPES-TALP+BSA before in vitro culture to avoid EV contamination. At 8 dpi, the blastocyst percentage out of presumed zygotes was scored and the ratio of hatching or hatched blastocysts were compared with the total number of blastocysts. Embryo quality was assessed by differential staining as explained before. The medium that supported embryo development and quality parameters the best, compared to our standard BSA medium, was selected for further experimental use. 

### 4.10. Experimental Design: Assessing the Functionality of Released EVs on Embryo Development and Quality 

In order to verify if enhanced bovine embryo development and embryo quality in group culture is associated with EV release, individual embryo culture was performed using EV enriched BSA embryo culture medium. This experiment was performed after determining that BSA macromolecule is not a source of contaminating EVs. As mentioned above, 1 mL pooled embryo conditioned medium was used for EV isolation by OptiPrep™ density gradient ultracentrifugation. The protein concentration of the isolated EV pellet, measured by Nanodrop™ (ND-100 spectrophotometer A280 nm), was 2.28 mg/mL after performing a first trial run with SOF+ITS+BSA medium containing three different protein concentrations (High: 22.8 µg/mL, Medium: 2.28 µg/mL, Low: 0.228 µg/mL). As mentioned above, we continued with a low EV protein concentration (0.228 µg/mL) since both higher protein concentrations were toxic for in vitro cultured bovine embryos. Subsequently, presumed zygotes (*n* = 989, 4 replicates) were allocated to four different groups (group culture control, individual culture control, individual culture control + PBS and EV enriched individual culture), culture medium was prepared and subsequent embryo culture was performed as described above. In order to test if EVs play a functional role in bovine embryo development in group, embryo development was evaluated on 7 dpi and 8 dpi while embryo quality was assessed by differential staining on 8 dpi.

### 4.11. Statistical Analysis

Statistical analyses were performed using R (R Core Team. R: A language and environment for statistical computing. R Foundation for Statistical Computing. Vienna, Austria. 2017). Normality of the variables was checked using the Shapiro–Wilk and the Kolmogorov–Smirnov test (*p* < 0.05). The effect of protein supplementation (BSA/PVP) and ultracentrifugation (UC BSA/UC PVP) of the media on cleavage rate, blastocyst yield (on day 7 and day 8) and, hatching rate was tested using an ANOVA test. Pairwise comparisons were done using Tukey’s post hoc test. The effect of protein supplementation and ultracentrifugation of the media on total cell number, trophectoderm and inner cell mass cell number, inner cell mass ratio and apoptotic cell ratio was tested using a Kruskal–Wallis test. Pairwise comparisons were done using Dunn’s post hoc test. Significance was declared at *p* < 0.05. The effect of EV supplementation during individual embryo culture on cleavage rate, blastocyst yield (on day 7 and day 8), hatching rate, total cell number, trophectoderm, inner cell mass, inner cell mass ratio and apoptotic cell ratio was tested using a Kruskal–Wallis test. Pairwise comparisons were done using Dunn’s post hoc test. Significance was declared at *p* < 0.05. Results are presented as mean and standard deviation.

### 4.12. Compliance with Ethics Requirements

All Institutional and National Guidelines for the care and use of animals (fisheries) were followed.

## 5. Conclusions

The findings described herein may shed some more light on the role that EVs are playing in embryo–embryo communication. Optimizing bovine embryo culture conditions towards a medium suitable for EV isolation will reduce the possible risk of contaminants that may interfere with EV quantification procedures. A major challenge to isolate EVs from embryo conditioned culture medium without contamination by other nanoparticles was overcome by a reliable EV isolation technique called OptiPrep™ density gradient ultracentrifugation. EV rich yields were present in OptiPrep™ density gradient fraction 8–9. Demonstrating that embryos-derived EVs were internalized by embryonic cells provides strong evidence for embryo–embryo crosstalk. After a possible EV contaminating effect of BSA in the embryo culture medium was ruled out, single embryo culture in EV-enriched BSA culture medium ultimately demonstrated increased blastocyst development and lower apoptotic cell ratios compared to similar embryo culture conditions lacking EVs. These observations provide strong evidence that enhanced bovine embryo development in group culture conditions is at least partly due to EVs that act as autocrine embryotropins in embryo–embryo communication during embryo culture. Follow-up research should focus on what kind of cargo is carried by these EVs, and whether this cargo differs based on the quality of embryos. Additionally, studies to evaluate whether EVs can also improve the pregnancy outcomes after transferring embryos, which were co-incubated with EVs during early embryo development, making them potential tools for the application of other biotechnologies. It would be also of interest to verify the biological action of EVs isolated from other reproductive fluids such as uterine, oviductal, and follicular fluid during embryo culture and to check if the enrichment of embryo culture media with these alternative sourced EVs support ‘single’ embryo culture more satisfactorily under in vitro conditions. In this respect, the use of EVs can also open new perspectives for optimization of assisted reproductive technologies in human medicine.

## Figures and Tables

**Table 1:** Blastocyst development and hatching rate of bovine embryos cultured in BSA and PVP media

Macromolecular Source	Ultra-centrifugation ^A^	No. of Presumed Zygotes	Cleavage Rate (%)	Blastocyst Yield (%)		
			Day 2	Day 7	Day 8	Hatching ^B^
BSA	No	625	86.9 ± 6.39	31.5 ± 5.27 ^a^	40.7 ± 3.39 ^a^	25.3 ± 7.73 ^a^
	Yes	450	88.2 ± 5.95	30.0 ± 5.96 ^ab^	40.7 ± 6.61 ^a^	25.6 ± 13.07 ^a^
PVP	No	515	88.0 ± 1.39	28.8 ± 4.04 ^ab^	38.6 ± 6.91 ^ab^	15.5 ± 6.83 ^b^
	Yes	309	87.3 ± 4.82	22.9 ± 3.88 ^b^	31.1 ± 4.63 ^b^	10.2 ± 8.46 ^b^

Data are represented as mean ± SD. ^A^ Culture media (SOF+ITS+BSA, SOF+ITS+PVP) were ultracentrifuged at 100,000× *g* for 18 h at 4 °C and supernatant of the media (EV-free) was used for in vitro embryo culture. ^B^ Hatching rates are shown as percentages of hatching or hatched blastocysts at 8 dpi compared with the total number of blastocysts. ^a, b^ Within each column, values that differ significantly are indicated by different superscripts (*p* < 0.05).

**Table 2:** Quality assessment of embryos cultured in BSA and PVP

Macromolecular Source	Ultra-centrifugation ^A^	No. of Embryos	Cell Number			ICM Ratio (%)	ACR (%)
			TCN	TE	ICM		
BSA	No	114	153 ± 4.89 ^a^	111 ± 4.02 ^a^	42 ± 3.17 ^a^	27.9 ± 1.73 ^a^	5.9 ± 0.45 ^a^
	Yes	114	139 ± 4.33 ^b^	101 ± 3.15 ^b^	38 ± 1.73 ^b^	27.5 ± 0.55 ^a^	8.7 ± 0.58 ^b^
PVP	No	103	120 ± 5.47 ^c^	89 ± 4.32 ^c^	31 ± 1.73 ^c^	27.3 ± 1.37 ^a^	12.4 ± 1.62 ^c^
	Yes	104	107 ± 6.35 ^d^	81 ± 5.48 ^c^	25 ± 1.42 ^d^	24.1 ± 1.50 ^b^	13.1 ± 1.70 ^c^

Data are represented as mean ± SD. ^A^ Culture media (SOF+ITS+BSA, SOF+ITS+PVP) were ultracentrifuged at 100,000× *g* for 18 h at 4° C and supernatant of the media (EV-free) was used for in vitro embryo culture. Total cell number (TCN) which includes the sum of both trophectoderm (TE) and inner cell mass cells (ICM), ICM ratio and apoptotic cell ratio (ACR) of blastocyst collected at 8 dpi were calculated after differential apoptotic staining. ^a–d^ Within each embryo quality parameter, values that differ significantly between culture media are indicated by different superscripts (*p* < 0.05).

**Table 3:** Blastocyst development and hatching rate of individual cultured bovine embryos after EV supplementation.

Treatment	System	No. of Presumed Zygotes	Cleavage Rate (%)	Blastocyst Yield (%)		
			Day 2	Day 7	Day 8	Hatching ^E^
Control ^A^	Group	225	82.2 ± 1.81 ^a^	36.8 ± 1.20 ^a^	49.7 ± 1.13 ^a^	25.0 ± 0.92 ^a^
Control ^B^	Individual	226	87.5 ± 0.11 ^a^	28.6 ± 0.06 ^b^	40.4 ± 0.08 ^b^	15.3 ± 0.87 ^b^
Control+PBS ^C^	Individual	232	89.3 ± 0.08 ^a^	32.9 ± 0.14 ^a^	40.7 ± 0.16 ^b^	14.8 ± 1.03 ^b^
EV supplemented ^D^	Individual	306	83.9 ± 0.08 ^a^	34.3 ± 0.09 ^a^	46.7 ± 0.10 ^a^	14.6 ± 1.09 ^b^

Data are represented as mean ± SD. ^A^ Control = SOF+ITS+BSA medium used in group culture system (25 presumed zygotes/50 µL droplet); ^B^ Control = SOF+ITS+BSA medium used in individual culture system (1 presumed zygotes/20 µL droplet); ^C^ Control+PBS = SOF+ITS+BSA medium with PBS (1:400); ^D^ EV supplemented = SOF+ITS+BSA medium supplemented with EVs in PBS with an EV concentration of 0.228 µg/mL. ^E^ Hatching rates are shown as percentages of hatching or hatched blastocysts at 8 dpi compared with the total number of blastocysts. ^a,b^ Within each column, values that differ significantly are indicated by different superscripts (*p* < 0.05).

**Table 4:** Quality assessment of individual cultured bovine embryos after EV supplementation.

Treatment	System	No. of Embryos	Cell Number			ICM Ratio (%)	ACN	ACR (%)
			TCN	TE	ICM			
Control	Group	36	120.8 ± 49.60 ^a^	84.7 ± 40.93 ^a^	36.1 ± 14.81 ^a^	31.0 ± 10.50 ^a^	7.14 ± 4.00 ^a^	5.9 ± 2.39 ^a^
Control^A^	Individual	36	100.0 ± 26.72 ^b^	67.4 ± 29.28 ^b^	32.5 ± 6.31 ^a^	35.5 ± 13.96 ^a^	7.6 ± 4.28 ^a^	8.3 ± 5.69 ^b^
Control+PBS ^B^	Individual	40	109.9 ± 30.63 ^a^	77.7 ± 29.79 ^a^	32.1 ± 6.28 ^a^	30.9 ± 9.42 ^a^	8.7 ± 4.41 ^a^	8.2 ± 4.24 ^b^
EV supplemented^C^	Individual	38	109.5 ± 33.30 ^a^	76.55 ± 32.66 ^a^	33.0 ± 5.65 ^a^	32.56 ± 10.32 ^a^	3.5 ± 2.91 ^b^	3.3 ± 2.90 ^a^

Data are represented as mean ± SD. ^A^ Control = SOF+ITS+BSA medium used in group culture system (25 presumed zygotes/50µL droplet); ^B^ Control = SOF+ITS+BSA medium used in individual culture system (1 presumed zygotes/20µL droplet); ^C^ Control+PBS = SOF+ITS+BSA medium with PBS (1:400); ^D^ EV supplemented = SOF+ITS+BSA medium supplemented with EVs in PBS with an EV concentration of 0.228µg/mL. Total cell number (TCN) which includes the sum of both trophectoderm (TE) and inner cell mass cells (ICM), apoptotic cell number (ACN), ICM ratio and, apoptotic cell ratio (ACR) of blastocyst collected at 8 dpi were calculated after differential apoptotic staining. ^a–c^ Within each column, values that differ significantly are indicated by different superscripts (*p* < 0.05).

## Supplementary Materials

Supplementary Materials can be found at http://www.mdpi.com/1422-0067/20/1/38/s1.

## Abbreviations

IVC	In vitro culture
FBS	Fetal bovine serum
EV	Extracellular vesicle
SOF	Synthetic oviductal fluid
BSA	Bovine serum albumin
PVP	Polyvinylpyrrolidone
UC	Ultracentrifugation
NTA	Nanoparticle tracking analytics
ITS	Insulin, transferrin, selenium
SOF+ITS+BSA	BSA medium
SOF+ITS+PVP	PVP medium
ODG	OptiPrep™ density gradient
CCM	Concentrated conditioned medium
hpi	Hours post insemination
dpi	Days post insemination
TCN	Total cell number
TE	Trophectoderm
ICM	Inner cell mass
ACN	Apoptotic cell number
ACR	Apoptotic cell ratio
PBS	Phosphate buffered saline
